# Antifungal susceptibility pattern of *Candida* species isolated from pregnant women

**DOI:** 10.3389/fcimb.2024.1434677

**Published:** 2024-08-07

**Authors:** Maqsood Ali, Wadhah Hassan Edrees, Wadee Abdullah Al-Shehari, Gao Xue, Safa Al-Hammadi, Eglal Ahmed Qasem, Ram Prasad Chaulagain, Nand Lal

**Affiliations:** ^1^ Department of Biochemistry and Molecular Biology, School of Basic Medicine, Harbin Medical University, Harbin, Heilongjiang, China; ^2^ Medical Microbiology Department, Faculty of Applied Science, Hajjah University, Hajjah, Yemen; ^3^ Medical Laboratory Department, Faculty of Medical Sciences, Al-Razi University, Sana’a, Yemen; ^4^ Medical Microbiology Department, Faculty of Medical Sciences, Ibb University, Ibb, Yemen; ^5^ Department Oral Medicine, Harbin Medical University, Harbin, Heilongjiang, China; ^6^ Department of Gastroenterology and Hepatology, Second Affiliated Hospital of Harbin Medical University, Harbin, Heilongjiang, China; ^7^ Department of Physiology, School of Biomedical Sciences, Harbin Medical University, Harbin, Heilongjiang, China

**Keywords:** antifungal, *Candida albicans*, pregnant women, vulvo vaginal candidiasis, Yemen

## Abstract

**Introduction:**

*Candida* species, opportunistic yeast, are the second most common cause of female vulvovaginal candidiasis. This study aimed to evaluate the antifungal susceptibility profile of the isolated *Candida* species in pregnant women in Hajjah governorate, Yemen.

**Methods:**

A hospital-based cross-sectional study was conducted among 396 pregnant women attending Authority AL-Gumhorri Hospital Hajjah between February and July 2023. Vaginal swabs were collected, and *Candida* species were isolated and identified based on the standard laboratory method. Furthermore, the antifungal drug susceptibility of *Candida* species was determined by the Kirby-Bauer technique.

**Results and discussion:**

The prevalence of vaginal *Candida* infection among pregnant women was 61.4%. *Candida albicans* was the most predominant species (59.26%), followed by *Candida krusei*(13.58%), *Candida Tropicalis* (11.12%), *Candida Grabata* (9.87%), and *Candida dubliniensis* (6.17%). The highest rate of *Candida* infections was among women aged 24–30 years (71.9%) who finished primary school (77.8%), with the third trimester (80%), multigravida (66.1%), and recurrent infection (67.7%) showing significant differences (P < 0.05). The *Candida albicans* isolates were resistant to clotrimazole and itraconazole at 34.7% and 23.6%, respectively.In addition, the resistance of *Candida krusei*, *Candida tropicalis*, *Candida glabrata*, and *Candida dublinensis* isolates to fluconazole, voriconazole, voriconazole, and nystatin was 57.6%, 63%, 43.8%, and 60%, respectively. Additionally, approximately 46.2% of isolated *Candida albicans* exhibited one kind of antifungal drug resistance, whereas 38.7% of isolated non-albicans exhibited resistance to three different antifungal agents. According to the above findings, *Candida* infection is highly prevalent in Yemen and quite widespread. Interventions in health education are advised to increase women’s knowledge of vaginitis and its prevention. The antifungal susceptibility test may also be helpful in determining the best medication for each patient.

## Introduction

Vulvovaginal candidiasis (VVC) commonly infects the mucous membrane of the lower genital tract in females. It is usually caused by *Candida albicans* but may occasionally be caused by other *Candida* species or yeasts. It is also known to have severe or persistent daily symptoms and is linked to an increased rate of antifungal resistance (Central for Diseases Control and Prevention (CDC), 2021).

Furthermore, it is common for *Candida* species, which are a part of the vulvovagina’s natural flora, to produce opportunistic infections when the host’s immune system is impaired. Because *Candida* sp. coexist in harmony with the vaginal microbiota, asymptomatic colonization is frequent and can last for years. Young women without symptoms have a genital *Candida* colonization rate of 20%, whereas pregnant women have a rate of up to 30% (Ghaddar et al., 2020; Sule-Odu et al., 2020).

Pruritus, vaginal discomfort, dyspareunia, external dysuria, and atypical vaginal discharge are typical symptoms of VVC. According to estimates, 75% of women will experience at least one episode of VVC, and 40%–45% of them may experience two or more episodes. VVC is characterized as both complicated and uncomplicated. A complicated VVC will affect 10%–20% of women, necessitating particular diagnostic and treatment considerations (Sule-Odu et al., 2020; Central for Diseases Control and Prevention (CDC), 2021).

The predominant species historically has been *Candida albicans*, which accounts for 85–95% of *Candida* vaginal infections. However, non-albicans *Candida* (NAC) species have been reported more frequently globally, with *Candida glabrata*, *Candida tropicalis*, *Candida parapsilosis*, *Candida krusei*, and *Candida dupliniesis* being the most common (Makanjuola et al., 2018; Sule-Odu et al., 2020).

A key risk factor is the age at which sexual engagement begins. Host-related factors include immunologic alterations, increased estrogen levels, diabetes mellitus, immunodeficiency states, antibiotic use, treatment with glucocorticoids, genetic predispositions, and behavioral factors such as birth control pills, intrauterine devices, spermicides, and condoms. Hygiene practices, tight-fitting clothing, and sexual behavior are thought to be risk factors linked to an increased rate of VVC (Disha and Haque, 2022).

The issue is particularly significant during pregnancy since *Candida* colonization is linked to infant mortality and premature delivery and because pregnant women can contaminate their unborn children to a degree of up to 65%, leading to invasive neonatal candidiasis. Due to the emergence of *Candida* species that are resistant to routinely used antifungal medications and repeated infections, the prevalence of vaginal candidiasis has recently substantially increased (Roberts et al., 2011; Masri et al., 2015).

Increased *Candida* species resistance to various antifungal treatments is a result of improper use of antifungal medications and a lack of adequate strategies to govern their usage, particularly in the treatment of vulvovaginal candidiasis. Azoles are the first-line medications for treating VVC initially, but fluconazole’s prolonged usage has led to the emergence of multidrug-resistant infections and recurring infections, which is a serious healthcare issue. To combat the medication resistance issue, it is urgently necessary to find novel compounds with antifungal capabilities due to various restrictions on the accessibility of current antifungal agents, ineffective therapy, high toxicity, low tolerability, and drug interaction (Lírio et al., 2019; Rosati et al., 2020; Nikoomanesh et al., 2023; Zaman et al., 2024).

One of the Yemeni governorates, Hajjah, lacks information on the antifungal susceptibility pattern of *Candida* species that cause vulvovaginal candidiasis in pregnant women. Therefore, the objective of this study was to evaluate the antifungal susceptibility profile of the isolated *Candida* species in pregnant women in Hajjah governorate, Yemen.

## Materials and methods

### Study design and period

**Figure 1:**
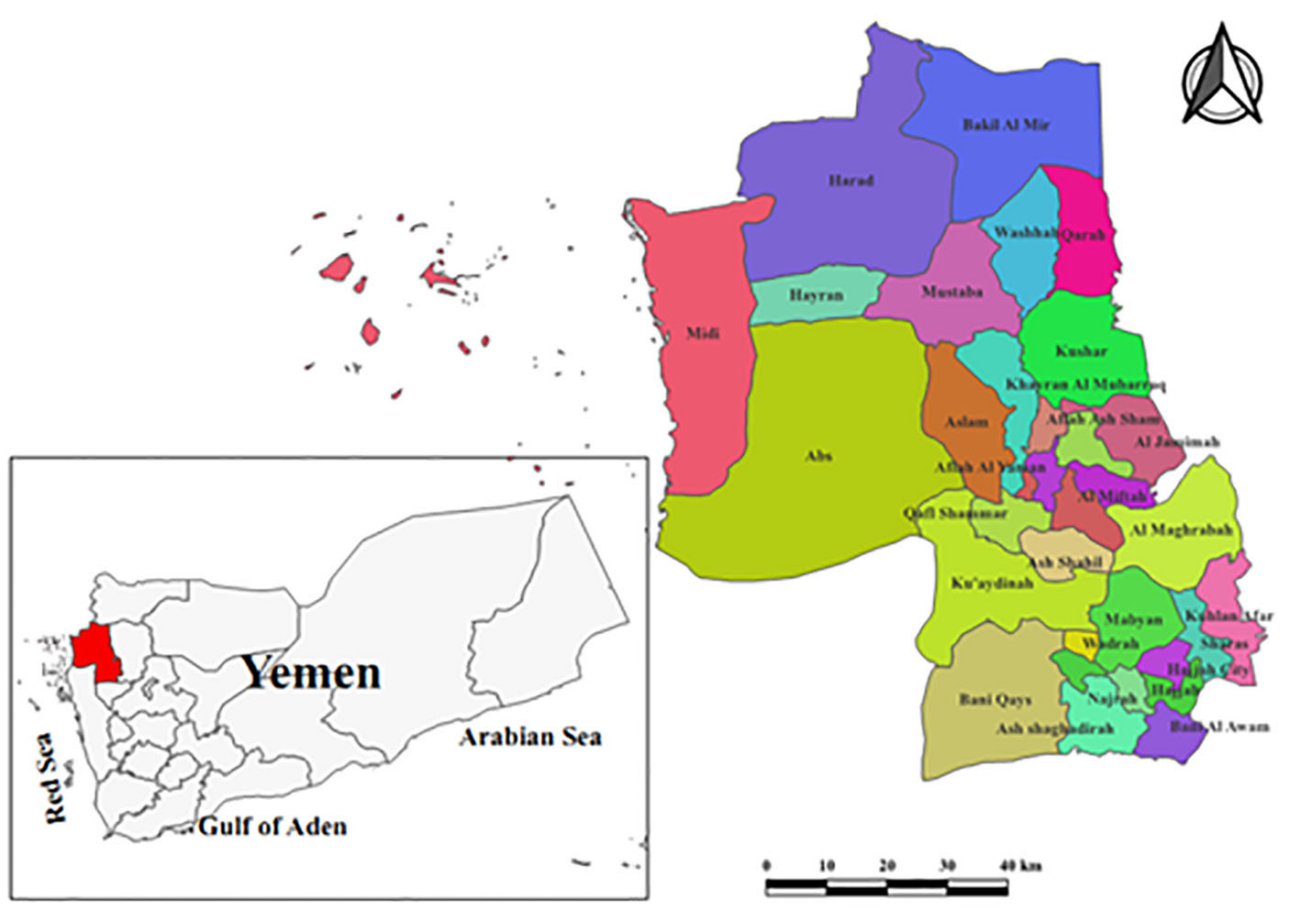
Map of Yemen showing study area in Hajjah governorate.

A hospital-based cross-sectional study was conducted in obstetrics and gynecology clinics at Authority Al-Gumhorri Hospital-Hajjah, which is situated in the Hajjah City. Hajjah is located in the northwest of Sana’a capital with a distance of 123 kilometers and an elevation of almost 1800 meters. Additionally, it is divided into 31 administrative districts on its border and consists of 2,630,678 inhabitants (**Figure 1**). The period of study was 6 months, from February to July 2023, and the laboratory analysis was performed at the hospital laboratory.

### Population size

The population size consisted of 396 pregnant women attending obstetrics and gynecology clinics with symptoms suggestive of vaginitis. The age of the participants ranged between 17 and 44 years, with an average age of 28.6 years.

### Data collection

A pretested questionnaire was developed based on multiple past studies (Khan et al., 2018; Abdul-Aziz et al., 2019), and some adjustments were made to gather the necessary data separately from each participant. Age, residence area, educational level, trimester, gravidity, frequency of infection, and medical signs and symptoms of vaginal abnormalities (burning, itching, and vaginal discharge) were all information that was included in the questionnaire. Research teams completed the questionnaire. The age categories were divided into four groups: 18–23 years, 24–30 years, 31–37 years, and 38–44 years.

### Inclusion and exclusion criteria

Women who gave vaginal specimens and had vaginal inflammation with discharge, burning, itching, and other symptoms during pregnancy were included. On the other hand, those who were currently receiving antimicrobial medicine treatment or refused to provide vaginal samples were not included in the study.

### Sample collection and examination

A well-qualified obstetrician collected vaginal swabs from each pregnant woman by using a sterile cotton swab. The swab was inserted approximately 5 cm into the vaginal opening, gently turned for approximately 30 seconds, and rubbed against the wall of the vagina. The collected specimens were labelled with patient information and sent directly to the medical laboratory for further examination (Mahon et al., 2015; Tille, 2017).

### Culture and identification of *Candida* species

The vaginal swabs were streaked individually under aseptic conditions onto Sabouraud Dextrose Agar (SDA) plate agar (HiMedia Lab., India) supplemented with chloramphenicol (250 mg/L). The inoculated plates were incubated aerobically at 37°C for 48 h. The growth colonies were inoculated onto HiCrome agar (HiMedia Lab., India) and incubated at 37°C for 48 h. In addition, the identification of *Candida* species was performed based on the *Candida* colony’s characterization of the medium. In addition, all isolates were further confirmed using the API 20C AUX strip, as recommended by the manufacturer (BioMereaux, Marcy-l`Étoile, France). The green colonies were identified as *C. albicans*, the purple colonies as *C. glabrata*, the pink colonies as *C. krusei*, the blue colonies as *C. tropicalis*, and the darker green colonies as *C. dublinensis*. Additionally, the germ tube test was used to distinguish between *C. albicans* and non-*albicans* (Mahon et al., 2015; Tille, 2017).

### Antifungal susceptibility testing

The disk diffusion test of the Kirby–Bauer technique was used to determine the antifungal drug susceptibility profiles of *Candida* species on Mueller Hinton agar (MHA) medium (HiMedia Lab., India) containing glucose (2%) and methylene blue dye (0.5 µg/mL) according to the guidelines of the Clinical and Laboratory Standards Institute (CLSI) (2021). A suspension of overnight cultures of *C. albicans, C. krusei, C. tropicalis, C. glabrata*, and *C. dublinensis* were prepared separately in 5 mL of sterile 0.85% saline. The turbidity containing 1-5×10^6^ CFU/mL of isolated *Candida* species was adjusted to McFarland 0.5 standard by using a DensiChek (*bioMerieux*) turbidity meter. A sterile cotton swab was moistened in the prepared suspension, and excess fluid was removed. Under aseptic conditions, the surface of Müller-Hinton agar (MHA) plate was streaked by the swab for three times and kept at room temperature for 15 minutes to dry. The antifungal discs, namely, amphotericin-B (50 µg), itraconazole (30 µg), fluconazole (25 µg), ketoconazole (10 µg), miconazole (30 µg), voriconazole (l0 µg), clotrimazole (10 µg), and nystatin (100 units) (HiMedia Lab., India), were placed over the surface of the MHA plate and incubated at 37°C for 48 h. According to CLSI (2021) recommendations, the zone of inhibition was measured in millimeters, and the results were interpreted as sensitive (S) and resistant (S) (**Table 1**) (Clinical and Laboratory Standards Institute (CLSI), 2021).

**Table 1:** Interpretative breakpoints of antifungal agents.

Antifungals	Disk concentration	Diameter of zone of inhibition (mm)	
		Sensitive	Resistant
Amphotericin-B	50 µg	≥15	≤12
Itraconazole	30 µg	≥22	≤18
Fluconazole	25 µg	≥39	≤29
Ketoconazole	10 µg	≥32	≤20
Miconazole	30 µg	≥26	≤22
Voriconazole	l0 µg	≥19	≤14
Clotrimazole	10 µg	≥20	≤11
Nystatin	100 units	≥ 15	≤10

### Quality control

The reference strains of *Candida albicans* ATCC 10231 and *Candida krusei* ATCC 6258 were used for quality control. In addition, the validity of the results was guaranteed through the implementation of standards of quality control during the pre-analytical, analytical, and post-analytical stages. All prepared culture media (plates and tubes) were checked for sterility and performance before being used for the isolation and identification of the studied *Candida* species. Positive controls included standard fungi and patient specimens. while negative control plates were un-inoculated. The positive and negative controls were employed to validate the quality of the isolation and identification results and to avoid the possibility of cross-contamination.

### Ethics approval

The Ethical Review Board of the Faculty of Applied Sciences at Hajjah University granted ethical clearance on January 24, 2023. Additionally, the Faculty of Applied Sciences presented a written document to the Authority Al-Gumhorri Hospital’s administration to approve the collection and analysis of specimens. Before collecting data and specimens, study volunteers were fully informed of the objectives and justification for their participation in the study. The informed consent form was obtained from educated individuals by taking their signatures, while the illiterate participants took a fingerprint instead of a signature. Additionally, the study’s confidentiality of data was maintained.

### Statistical analysis

The statistical analysis was performed using SPSS version 20 (Statistical Package for Social Science). Descriptive statistics, numbers, frequencies, percentages, and tables were used to describe the findings. Moreover, chi-square (χ2), confidence interval (95% CI), and odds ratio (OR) were used to assess the association between dependent and independent variables. Finally, a p value less than 0.05 was considered statistically significant.

## Results

This research included approximately 396 pregnant women. **Table 2** lists the participants’ characteristics, including the fact that 128 (32.3%) of them were between the ages of 24 and 30 years, 279 (70.5%) were from urban areas, 206 (52%) had completed their secondary education, 180 (45.5%) were in the third trimester, 207 (52.3%) were primigravida, and 279 (70.5%) were in the first time of infection.

**Table 2:** Sociodemographic characteristics of the pregnant women participating in the study.

Variables		Frequency	Rate (%)
Age (in years)	17-23	99	25.0
	24-30	128	32.3
	31-37	106	26.8
	38-44	63	15.9
Resident area	Urban	279	70.5
	Rural	117	29.5
Educational status	Illiterate	45	11.4
	Primary	81	20.5
	Secondary	206	52.0
	Graduate	64	16.2
Gestational trimester	1st trimester	90	22.7
	2nd trimester	126	31.8
	3rd trimester	180	45.5
Gravidity	Primigravida	207	52.3
	Multigravida	189	47.7
Frequency of infection	First time	117	29.5
	Recurrent	279	70.5

**Figure 2:**
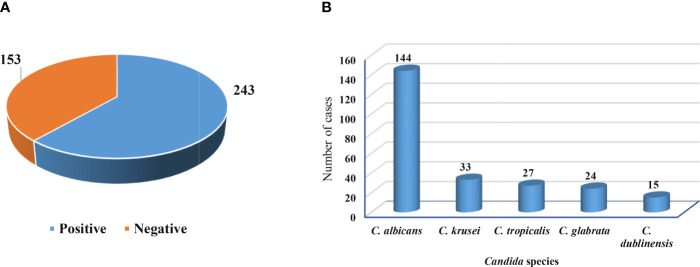
Growth in culture media and isolated *Candida* species. **(A)** Positive and negative growth of vaginal swabs in culture media. **(B)** Frequency of *Candida* species isolated from pregnant women.

**Figure 2A** shows that out of 396 vaginal swabs, 243 (61.4%) were positive for growth in culture media, while 153 (38.6%) were negative. Additionally, the frequency rates of isolates of *C. albicans* were 144 (59.26%) followed by *C. krusei* 33 (13.58%), *C. tropicalis* 27 (11.12%), *C. glabrata* 24 (9.87%), and *C. dublinensis* 15 (6.17%) as figured in **Figure 2B**.

According to this finding, the highest rate of *Candida* infections was recorded among pregnant women aged between 24 and 30 years (71.9%), and the lowest rate was among individuals aged 38–44 years (36.5%), with a statistically significant difference (95% CI =2.23–2.44; *P* = 0.000). Similarly, the participants who lived in urban areas had the highest rate (64.5%) compared with those who lived in rural areas (53.8%). Furthermore, the study subjects who completed primary school had a higher rate of *Candida* infections (77.8%), whereas the uneducated participants had a lower rate (40.0%), with a statistically significant difference (*P* = 0.000). Additionally, a high rate of infection was significantly observed among pregnant women in the third trimester (80.0%) compared to others, with a statistically significant difference (*P* = 0.004). Furthermore, the multigravida mothers were found to be more infected (66.1%) than primigravida participants (63.8%), with a statistically significant difference (*P* = 0.000). Moreover, recurrent infections showed a high rate among participants (67.7%; *P* = 0.117) compared to the first time of infection (46.2%), as listed in **Table 3**.

**Table 3:** Association between positive *Candida* infection and socio-demographic and risk factors.

Variables	No. (%)	Mean ± S.D	Positive No. (%)	Negative No. (%)	OR 95%CI	X	P value
Age (in years)							
17-23	99 (25)	2.34 ± 1.022	63 (63.6)	36 (36.4)	NA (2.23–2.44)	39.914	0.000
24-30	128 (32.3)		92 (71.9)	36 (28.1)			
31-37	106 (26.8)		65 (61.3)	41 (38.7)			
38-44	63 (15.9)		23 (36.5)	40 (63.5)			
Resident area							
Urban	279 (70.5)	1.70 ± 0.457	180 (64.5)	99 (35.5)	0.835 (1.66–1.75)	56.333	0.693
Rural	117 (29.5)		63 (53.8)	54 (46.2)			
Educational status							
Illiterate	45 (11.4)	2.73 ± 0.866	18 (40)	27 (60)	NA (2.64–2.82)	125.971	0.004
Primary	81 (20.5)		63 (77.8)	18 (22.2)			
Secondary	206 (52)		131 (63.6)	75 (36.4)			
Graduate	64 (16.2)		31 (48.4)	33 (51.6)			
Gestational trimester							
1st trimester	90 (22.7)	2.23 ± 0.795	36 (40)	54 (60)	NA (2.15–2.31)	78.000	0.000
2nd trimester	126 (31.8)		63 (50)	63 (50)			
3rd trimester	180 (45.5)		144 (80)	36 (20)			
Gravidity							
Primigravida	207 (52.3)	1.48 ± 0.500	132 (63.8)	75 (36.2)	1.086 (1.43–1.53)	1.815	0.000
Multigravida	189 (47.7)		125 (66.1)	63 (33.9)			
Frequency of infection							
First time	117 (29.5)	1.7045 ± 0.45682	54 (46.2)	63 (53.8)	0.681 (1.66–1.75)	75.000	0.117
Recurrent	279 (70.5)		189 (67.7)	90 (32.3)			

SD, Standard Division; χ^2^, Chi-square; CI, Confidence interval; OR, Odds ratio, P value (p< 0.05: significant); NA, Not Applicable.

The current research found that participants’ clinical signs and symptoms and *Candida* sp. prevalence were statistically significant (P < 0.05). Additionally, as shown in **Table 4**, pregnant women who experienced discharge (72.7%; OR= 2.667; 95% CI=1.21–1.29) and itching (64.5%; OR= 0.335; 95% CI=1.03–1.07) had the greatest proportion of *Candida* sp. positivity.

**Table 4:** Clinical signs and symptoms associated with positive *Candida* sp.

Variables	No.	Mean ± S.D	Positive	Negative	OR 95%CI	X	P value
Itching							
Yes	377 (95.2)	1.05 ± 0.214	243 (64.5)	134 (34.5)	0.355 (1.03–1.07)	NA	0.000
No	19 (4.8)		0 (0)	19 (100)			
Burning							
Yes	342 (86.4)	1.14 ± 0.344	198 (57.9)	144 (42.1)	0.695 (1.10–1.17)	96.333	0.003
No	54 (13.6)		45 (83.3)	9 (16.7)			
Discharge							
Yes	297 (75)	1.25 ± 0.434	216 (72.7)	81 (27.3)	2.667 (1.21–1.29)	147.00	0.000
No	99 (25.0)		27 (27.3)	72 (72.7)			

SD, Standard Division; χ^2^, Chi-square; CI, Confidence interval; OR, Odds ratio, P value (p< 0.05: significant); NA, Not Applicable.

**Figure 3:**
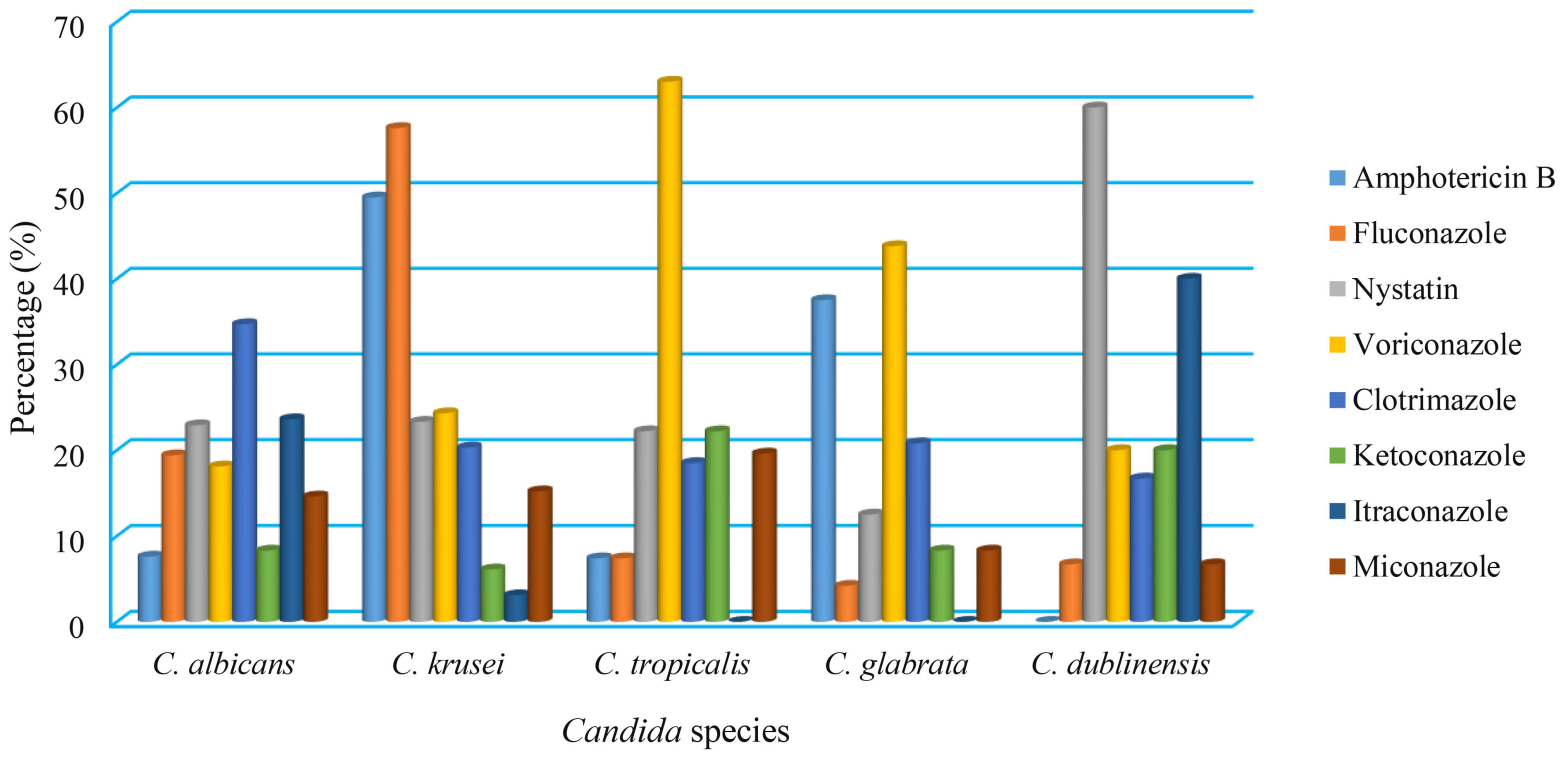
Antifungal resistant pattern of isolated *Candida* species.

According to the antifungal susceptibility data, *C. albicans* isolates were highly susceptible to amphotericin B, which had a 92.4% sensitivity rate, followed by ketoconazole (91.7%), miconazole (85.4%), voriconazole (81.9%), and fluconazole (80.6%). In contrast, clotrimazole and itraconazole were both ineffective against 34.7% and 23.6% of *C. albicans* isolates, respectively. In addition, the *C. krusei*, *C. tropicalis*, *C. glabrata*, and *C. dublinensis* isolates were resistant to fluconazole (57.6%), voriconazole (63%), voriconazole (43.8%), and nystatin (60%), respectively, (**Table 5**, **Figure 3**).

**Table 5:** Antifungal susceptibility pattern of isolated *Candida* species.

Antifungal drug	*C. albicans* (n=144)		*C. krusei* (n=33)		*C. tropicalis* (n=27)		*C. glabrata* (n=24)		*C. dublinensis* (n=15)	
	S No. (%)	R No. (%)	S No. (%)	R No. (%)	S No. (%)	R No. (%)	S No. (%)	R No. (%)	S No. (%)	R No. (%)
Amphotericin B	133 (92.4)	11 (7.6)	17 (51.5)	15 (49.5)	25 (92.6)	2 (7.4)	15 (62.5)	9 (37.5)	15(100)	0 (0)
Fluconazole	116 (80.6)	28 (19.4)	14 (42.4)	19 (57.6)	25 (92.6)	2 (7.4)	23 (95.8)	1 (4.2)	14 (93.3)	1 (6.7)
Nystatin	111 (77.1)	33 (22.9)	22 (66.7)	11 (23.3)	21 (77.8)	6 (22.2)	21 (87.5)	3 (12.5)	6 (40.0)	9 (60.0)
Voriconazole	118 (81.9)	26 (18.1)	25 (75.7)	8 (24.3)	10 (37.0)	17 (63.0)	13 (54.2)	11 (43.8)	12 (80.0)	3 (20.0)
Clotrimazole	94 (65.3)	50 (34.7)	23 (79.7)	10 (20.3)	22 (81.5)	5 (18.5)	19 (79.2)	5 (20.8)	11 (73.3)	4 (16.7)
Ketoconazole	132 (91.7)	12 (8.3)	31 (93.9)	2 (6.1)	21 (77.8)	6 (22.2)	22 (91.7)	2 (8.3)	12 (80.0)	3 (20.0)
Itraconazole	110 (76.4)	34 (23.6)	32 (96.9)	1 (3.1)	27 (100)	0 (0)	24 (100)	0 (0)	9 (60.0)	5 (40.0)
Miconazole	123 (85.4)	21 (14.6)	28 (84.8)	5 (15.2)	19 (70.4)	8 (19.6)	22 (91.7)	2 (8.3)	14 (93.3)	1 (6.7)

S, sensitive; R, Resistant.

Of the nine tested antifungal drugs, the high-sensitivity proportion of isolated *Candida* sp. was to ketoconazole at 218 (89.7%), while the highest rate of *Candida* species resistance was for clotrimazoleat 74 (30.5%), followed by Voriconazole 65 (26.7%), Nystatin 62 (25.5%), and lowest resistant was for Ketoconazole 25 (10.3%) (**Table 6**).

**Table 6:** Total susceptibility pattern of isolated *Candida* species.

Antifungal drug	Sensitive		Resistant	
	No.	%	No.	%
Amphotericin_B	206	84.8	37	15.2
Fluconazole	192	79.0	51	21.0
Nystatin	181	74.5	62	25.5
Voriconazole	178	73.3	65	26.7
Clotrimazole	169	69.5	74	30.5
Ketoconazole	218	89.7	25	10.3
Itraconazole	203	83.5	40	16.5
Miconazole	206	84.8	37	15.2

According to **Table 7**, the majority of isolated *Candida albicans* (46.2%) exhibited only one kind of antifungal drug resistance, compared to 38.7% of isolated non-albicans *Candida*, which exhibited resistance to three different antifungal drug types (38.7%).

**Table 7:** Multiple-antifungal resistance profiles of *Candida* isolates.

Species	No. (%)	Antibiogram patterns				
		R1	R2	R3	R4	R5
*C.albicans*	144 (59.3)	54 (46.2%)	37 (31.6%)	20 (17.1%)	4 (3.4%)	2 (1.7%)
Non-albicans *Candida*	99 (40.7)	23 (30.7%)	14 (18.7%)	29 (38.7%)	8 (10.7%)	1 (1.3%)
Total	243 (100)	77 (31.68%)	51 (20.98%)	49 (20.16%)	12 (4.94%)	3 (1.23%)

R1/R2/R3/R4/R5, Resistant to one/two/three/four/five antifungal agents.

## Discussion

The present finding revealed that the prevalence proportion of *Candida* sp. was 61.4% among pregnant women, which is close to the finding reported among pregnant women in Ibb City (Edrees et al., 2020). This outcome is less than a report conducted in Kenya, which found that 90.38% of pregnant women were infected by *Candida* sp (Nelson et al., 2013). In contrast, the current finding is lower than several reports that showed that the prevalence rate of *Candida* species among pregnant women was 55.4% in Cameroon (Toua et al., 2013), 60.8% in Egypt (Abbas et al., 2016), 51.6% in Sana’a, Yemen (Al-Rukeimi et al., 2020), 34% in Saudi Arabia (Venugopal et al., 2021), and 25% in north-western Ethiopia (Tsega and Mekonnen, 2019). The great variation in frequency rate might be due to the differences in geographical locations, study population, sample size, hygienic conditions, socioeconomic status, and diagnostic methods employed by the participants.

The present study revealed that the frequency rates of *C. albicans*, *C. krusei*, *C. tropicalis*, *C. glabrata*, and *C. dublinensis* were reported to be 59.26%, 13.58%, 11.12%, 9.87%, and 6.17%, respectively. This result is similar to some reports performed in different countries (Tsega and Mekonnen, 2019; Ghaddar et al., 2020; Venugopal et al., 2021). The widespread use of antifungal drugs over-the-counter, inappropriate use, incomplete description of treatment, longer treatment for recurrent candidiasis, and use of effective agents to eliminate *C. albicans* have all been proposed as potential explanations for the increased isolation of non-albicans *Candida* species from vulvovaginitis patients. Given the high frequency of VVC among women in the study area, it is important to screen or treat pregnant women for VVC to prevent its detrimental health effects (Sule-Odu et al., 2020; Zaman et al., 2024).

Remarkably, the highest rate of *Candida* infections in this study was noticed in pregnant women aged between 24 and 30 years (71.9%). Similar reports documented that a high frequency of *Candida* species was found at 60% among the ages 26–35 (Nelson et al., 2013), 38.5% in the ages 34–40 (Tsega and Mekonnen, 2019), 37.4% in the ages 20–34 (Konadu et al., 2019), and 44.4% in the ages 26–35 (Waikhom et al., 2020).

The cause of this might be explained by the fact that women in this age range release many reproductive hormones, which can inhibit the immune system and foster *Candida* infection. The use of antibiotics, which kill bacteria, including natural flora, is another factor that might be involved. This will give *Candida* a chance to attack the vaginal wall (Tsega and Mekonnen, 2019). Additionally, this high proportion is caused by the vagina’s higher glycogen content and high estrogen hormone levels. It offers a reliable source of carbon, which helps with *Candida* proliferation.

The present outcome showed that the highest rate of *Candida* infections was recorded among those living in urban areas (64.5%). The outcome, consistent with Abdul-Aziz et al. (Abdul-Aziz et al., 2019), revealed that the highest distribution of VVC was among pregnant women in the urban area, at 88.44%. The prevalence of the infection was higher in uneducated women than in patients with basic school education and above, and there was a statistically significant correlation between vulvovaginal candidiasis and educational level (*P* = 0.004). This finding was compatible with earlier reports (Konadu et al., 2019; Waikhom et al., 2020) that revealed a high frequency of *Candida* sp. among those with basic education. The difference in infection rates between illiterate individuals and those with more education could be explained by improvements in personal cleanliness and/or economic position brought on by education (Bitew and Abebaw, 2018).

Compared to primigravidae (63.8%), multigravidae (66.1%) women exhibited a higher rate of *Candida* colonization, with significant differences (*P* = 0.000) in the present study. Similarly, research conducted in Pakistan found that multigravidae women experienced the condition more frequently than primigravidae women, with results of 60% and 40%, respectively (Aslam et al., 2008). Further research from Nepal (Shrestha et al., 2011) supports our findings. The rationale is that the rate of infection rises with the frequency of pregnancies (3^rd^> 2^nd^> 1^st^), which lowers immunity and may lead to extensive *Candida* colonization. Additionally, a study by Tsega and Mekonnen (2019) showed that *Candida* infection was more common among women with multigravidae (61.5%) than among those with primigravidae (38.5%).

According to these data, females with recurrent infections had a greater prevalence rate of vulvovaginal candidiasis (67.7%) than those who had it for the first time (29.5%). This result conflicts with research performed in Nigeria by Sobel (2007). The significantly rising rate of recurrent vaginal candidiasis infections in this research may be due to an increase in *Candida* species that are resistant to widely used antifungal medications.

The current results revealed that there were significant differences between the gestational period and *Candida* colonization (80%; P = 0.000). The highest rate of vaginal *Candida* species was detected in the third-trimester participants at 80%, while the lowest was in the first trimester (40%). A similar finding was further reported: the highest rate of *Candida* species was in the third trimester, at 68.09% in Kenya (Nelson et al., 2013) and 57.4% in Ghana (Waikhom et al., 2020). According to research, third-trimester pregnant women were most likely to develop an illness. Pregnancy raises the risk of VVC, and the risk might reach 50% during the final trimester (Konadu et al., 2019; Tsega and Mekonnen, 2019; Waikhom et al., 2020).

This is because during pregnancy, particularly in the third trimester, elevated estrogen levels generate larger glycogen stores in the vagina, which serve as an excellent source of carbon and encourage the growth of *Candida* sp. Additionally, estrogen makes vaginal epithelial cells’ yeast cytosol receptors more attractive to *Candida* species (Babić and Hukić, 2010). So, to prevent vaginal microorganisms’ infections, particularly in the last trimester, it is imperative that health education interventions be enhanced and women’s awareness raised about the disease (Abruquah, 2012; Holzer et al., 2017).

The current findings showed that 92.4% of *C. albicans* were sensitive to amphotericin B. This finding was similar to reports that recorded 93.8% in Ethiopia (Tsega and Mekonnen, 2019) and 87.2% in Ghana (Kan et al., 2023). Amphotericin-B is more sensitive than other antibiotics because it is not frequently given and used extensively due to its high cost, difficulties in administration, and severe renal toxicity. Therefore, the less a drug is used, the less likely it is to develop resistance to it (Tsega and Mekonnen, 2019). Consequently, to avoid the emergence and spread of drug-resistant bacterial strains, it is critical to conduct periodic surveillance of antimicrobial susceptibility testing and proper management of pregnant women (Abruquah, 2012).

The overall rate of *Candida* species in the present investigation was highly resistant to clotrimazole (30.5%), voriconazole (26.7%), nystatin (25.5%), and fluconazole (21.0%). These results are lower than the results of Khan et al (Khan et al., 2018), who detected a high resistance rate of *Candida* sp. against fluconazole (62%), clotrimazole (59.3%), itraconazole (40.7%), and voriconazole (10.2%). Another study by Tsega and Mekonnen (2019) revealed that a high resistance rate of *Candida* sp. (57.3%) was found for ketoconazole and itraconazole. In addition, approximately 17.2% and 5.7% of *Candida* species were resistant to fluconazole and flucytosine, respectively (Bitew and Abebaw, 2018). Hence, more research on family health strategies is required for developing a better understanding of how drug-resistant microbes spread and their role in health-related infections.

The current study showed that ketoconazole was the most effective antifungal drug when compared to tested antifungal agents that had 89.7% effectiveness. In different reports, Bitew and Abebaw (2018) reported that fluconazole was the most effective antifungal drug, while Tsega and Mekonnen (2019) documented clotrimazole as an effective antifungal agent. Earlier studies demonstrated that the resistance rate in *Candida* sp. to voriconazole and fluconazole has remained constant over a decade (Pfaller et al., 2007; Lyon et al., 2010; Bitew and Abebaw, 2018). According to Gualco et al., (2007), *C. albicans* exhibited resistance to fluconazole and itraconazole at rates of 0.7% and 2.7%, respectively. To determine the most effective antibiotic, establishing a national antibiotic policy is crucial for regulating the administration of antibiotics to patients prior to conducting an antibiotic sensitivity test, which aims to identify the most optimal antibiotic.

Furthermore, the isolated *C. krusei*, *C. tropicalis*, *C. glabrata*, and *C. dublinensis* in this finding were resistant to fluconazole (57.6%), voriconazole (63%), voriconazole (43.8%), and nystatin (60%), respectively. A report by Luo et al. (Luo et al., 2016) found that the resistance levels of *C. albicans*, *C. glabrata*, and *C. tropicalis* to amphotericin B, voriconazole, fluconazole, 5-fluorocytosine, and itraconazole were found to be 0.5% to 6.4%, 0% to 7.7%, and 0% to 9.6%, respectively. In addition, itraconazole (24.1%) and posaconazole (14.5%) were the only two drugs for which resistance rates were noted to *C. glabrata*. Whereas *C. krusei* exhibited the highest resistance rates to itraconazole at 81.5% (Peman et al., 2012). Variations in the research populations and geographic settings, as well as variations in the usage of antifungals, could be an explanation for the varying resistance rates observed in this investigation.

According to the multidrug resistance results, a high proportion of *Candida albicans* (46.2%) were resistant to one type of antifungal drug, while 38.7% of isolated non*-albicans Candida* were resistant to three types of antifungal drugs. A study by Tsega and Mekonnen (2019) revealed that 46.29% of isolated *C. albicans* were resistant to three types of antifungal agents, and non*-albicans *Candida**, including *C. glabrata* (35.29%) and *C. krusei* (57.17%), were resistant to one type of antifungal. Therefore, healthcare settings should strongly consider implementing an antibiogram as part of their infection control programs to guide their decision-making on appropriate empirical treatment and infection control programs (Brandão et al., 2018).

## Strength and limitation of the study

This study is the first to try to shed some light on the prevalence of *Candida* species and their antifungal susceptibility profiles among pregnant women in Hajjah governorate, Yemen, which is an important public health issue that has not been previously studied in this region. Additionally, the results of this investigation are expected to yield valuable insights that will provide decision makers with antifungal susceptibility profiles in the study area and encourage other researchers to conduct further studies in this field. However, the limitations of this work include the fact that modern and advanced techniques such as polymerase chain reaction (PCR) for genotypes identifying the isolated *Candida* species are lacking due to limited resources. In addition, this study is unable to cover the behavioral risk factors that may be associated with the prevalence of *Candida* species among study participants.

## Conclusion

The greater frequency of *Candida* species isolates in this study is considered a serious health problem for pregnant women. The high humidity in Hajjah may have contributed to the spread of *Candida* species among women. Therefore, to increase women’s knowledge of vaginal candidiasis and its prevention, health education interventions are highly recommended. Furthermore, screening women for vaginal infections prior to treatment is recommended, as well as educating spouses and partners on the transmission and prevention of sexually transmitted illnesses. The consequences of candidiasis can be avoided with proper and immediate treatment and diagnosis. Moreover, the antifungal susceptibility test may help determine the best drug therapy for each patient. Accordingly, future investigations should focus on the occurrence of drug-resistant *Candida* strains and their emergence.

## Data availability statement

The raw data supporting the conclusions of this article will be made available by the authors, without undue reservation.

## Ethics statement

The studies involving humans were approved by Authority AL-Gumhorri Hospital Hajjah. The studies were conducted in accordance with the local legislation and institutional requirements. Written informed consent for participation in this study was provided by the participants’ legal guardians/next of kin.

## Author contributions

MA: Writing – original draft, Writing – review & editing, Conceptualization, Data curation, Formal analysis, Investigation, Methodology, Funding acquisition, Project administration, Resources, Software, Supervision, Validation, Visualization. WE: Conceptualization, Data curation, Writing – review & editing, Methodology, Formal analysis, Resources, Validation. WA-S: Investigation, Resources, Software, Writing – review & editing. GX: Writing – review & editing, Supervision. SH: Writing – review & editing, Formal analysis, Software. EQ: Writing – review & editing, Resources. RC: Investigation, Visualization, Writing – review & editing, Software. NL: Writing – review & editing, Data curation, Investigation, Visualization.
